# Genome-wide characterization, functional analysis, and expression profiling of the *Aux/IAA* gene family in spinach

**DOI:** 10.1186/s12864-024-10467-z

**Published:** 2024-06-05

**Authors:** Erfan Imani Asl, Aboozar Soorni, Rahim Mehrabi

**Affiliations:** Department of Biotechnology, College of Agriculture, Isfahan University of Technology, Isfahan, Iran

**Keywords:** *Aux/IAA*, Spinach, Gene identification, Phylogenetic analysis, Gene expression

## Abstract

**Background:**

The auxin/indole-3-acetic acid (Aux/IAA) gene family is a crucial element of the auxin signaling pathway, significantly influencing plant growth and development. Hence, we conducted a comprehensive investigation of *Aux/IAAs* gene family using the Sp75 and Monoe-Viroflay genomes in spinach.

**Results:**

A total of 24 definitive *Aux/IAA* genes were identified, exhibiting diverse attributes in terms of amino acid length, molecular weight, and isoelectric points. This diversity underscores potential specific roles within the family, such as growth regulation and stress response. Structural analysis revealed significant variations in gene length and molecular weight. These variations indicate distinct roles within the *Aux/IAA* gene family. Chromosomal distribution analysis exhibited a dispersed pattern, with chromosomes 4 and 1 hosting the highest and lowest numbers of *Aux/IAA* genes, respectively. Phylogenetic analysis grouped the identified genes into distinct clades, revealing potential evolutionary relationships. Notably, the phylogenetic tree highlighted specific gene clusters suggesting shared genetic ancestry and potential functional synergies within spinach. Expression analysis under NAA treatment unveiled gene-specific and time-dependent responses, with certain genes exhibiting distinct temporal expression patterns. Specifically, SpoIAA5 displayed a substantial increase at 2 h post-NAA treatment, while SpoIAA7 and SpoIAA9 demonstrated continuous rises, peaking at the 4-hour time point.

**Conclusions:**

These observations indicate a complex interplay of gene-specific and temporal regulation in response to auxin. Moreover, the comparison with other plant species emphasized both shared characteristics and unique features in *Aux/IAA* gene numbers, providing insights into the evolutionary dynamics of this gene family. This comprehensive characterization of *Aux/IAA* genes in spinach not only establishes the foundation for understanding their specific functions in spinach development but also provides a valuable resource for experimental validation and further exploration of their roles in the intricate network of auxin signaling pathways.

## Background

Auxin, a pivotal phytohormone, plays a central role in orchestrating fundamental processes throughout plant development. Its multifaceted influence spans various developmental stages across the entirety of the plant life cycle [1–6]. The key players in the intricate pathway of auxin signaling are the auxin/indole-3-acetic acid (*Aux/IAA*) genes. This family of genes, recognized as primary auxin-responsive entities, has been pivotal in unraveling the molecular intricacies of plant growth regulation [7, 8]. These proteins play a dual role in the orchestration of auxin signaling. Under low-auxin conditions, Aux/IAA proteins repress the activity of Auxin Response Factor (ARF) transcription factors. However, an increase in auxin levels triggers the rapid degradation of Aux/IAA proteins, thereby de-repressing ARF activity and facilitating a cascade of auxin-mediated transcriptional changes [9–11]. Aux/IAA proteins, pivotal in the auxin signaling cascade, exhibit a distinctive four-domain structure, with each domain intricately contributing to their functionality. Domain I, located at the N-terminus, is distinguished by a leucine repeat motif commonly denoted as the ‘LxLxL’ motif. This motif serves as a repressor domain, playing a crucial role in transcriptional control and regulation [11]. In contrast, domain II, marked by its high conservation, emerges as a linchpin in the delicate balance of protein stability. Mutations in domain II can significantly impact the half-life of Aux/IAA proteins, influencing their rapid degradation under basal conditions [12]. Domains III and IV, the less-explored but equally significant constituents of Aux/IAA proteins, enhance the multifaceted roles of these proteins. Domain III is predicted to form a genuine protein structural domain that can independently fold into a βα α structure. This structural feature implies its potential sufficiency for dimerization, a phenomenon crucial for the interaction of Aux/IAA proteins with each other and ARF proteins [11, 13]. Furthermore, Domain IV, positioned at the C-terminus, is known to harbor a functional Nuclear Localization Sequence (NLS) and likely contributes to dimerization processes [14, 15]. The intricate interplay between these four domains defines the dynamic nature of Aux/IAA proteins in response to auxin stimuli. While Domain I governs transcriptional repression, Domain II ensures the swift degradation of these proteins. Domains III and IV, through their structural and functional characteristics, add an additional layer of complexity, orchestrating the dimerization events critical for the subtle regulatory actions of Aux/IAA proteins.

Evolutionary insights into *Aux/IAA* genes have been investigated through genomic studies. Phylogenetic analyses could trace the origins of *Aux/IAA* genes, illuminating the ancient roots of auxin signaling [16]. Positive selection in flowering plants, as evidenced by the Ka/Ks ratios among auxin signaling genes, underscores their adaptive evolution and essential role in complex auxin responses [17]. While functional studies in model plants like Arabidopsis have highlighted the diverse roles of *Aux/IAA* genes in plant development, the details of their regulatory networks and functional redundancies remain intriguing [18]. The genetic redundancy among family members, often leading to a scarcity of loss-of-function phenotypes, prompts further exploration into the subtle roles and interplay of Aux/IAA proteins in plant biology. Besides, advancements in genomic technologies have facilitated the comprehensive exploration of the *Aux/IAA* gene family across diverse plant species. Studies in model plants such as *Arabidopsis thaliana* [19], rice [20], tomato [21], maize [22], and non-model plants such as sorghum [23], poplar [24], *Salix suchowensis* [25], and *Prunus mume* have contributed to the identification and annotation of *Aux/IAA* genes within their respective genomes. Notably, the availability of complete genome sequences, as exemplified in cucumber [26], has been instrumental in advancing our understanding of the entire *Aux/IAA* gene repertoire [27].

Spinach (*Spinacia oleracea*) is a widely cultivated leafy green vegetable known for its nutritional value and culinary versatility. Despite its agricultural importance, there is a lack of information regarding the comprehensive characterization and functional analysis of the *Aux/IAA* gene family in spinach. In light of the pivotal roles played by genes involved in auxin signaling in plant growth and development, this study aims to address the following objectives: (1) Systematic identification of *Aux/IAA* genes in the spinach genome. (2) Characterization of their sequence features, including conserved domains and cis-regulatort elements. (3) The genomic distribution analysis of *Aux/IAA* genes in the spinach genome. (4) Examination of the evolutionary relationships among these genes to understand their origin and divergence in land plants. (5) Expression Profiling of *Aux/IAA* genes in various tissues and at different developmental stages of spinach.

This research is unique and novel compared to existing studies on *Aux/IAA* genes in other plants as it represents the first comprehensive analysis of the *Aux/IAA* gene family specifically in spinach. Unlike previous studies, which have primarily focused on model organisms such as Arabidopsis, our investigation extends to spinach and offers a comparative perspective with other vegetative species such as radish and lettuce. By employing advanced genomic and bioinformatics techniques, this study provides new insights into the sequence characteristics, genomic distribution, and evolutionary history of *Aux/IAA* genes in spinach.

## Results

### Identification of the *Aux/IAA* gene family in spinach genome

In the present investigation, we undertook a meticulous approach to identify and characterize *Aux/IAA* genes in spinach. Initially, a set of 35 candidate genes was identified using HMM. Subsequent refinement through the SMART online tool, targeting the exclusion of genes containing the B3 domain, led to the confirmation of 24 definitive *Aux/IAA* genes. Detailed analyses of these genes were then conducted, encompassing crucial parameters such as amino acid length, molecular weight, and isoelectric point (Table 1). The results, presented in Table 1, elucidate the diverse characteristics of the identified *Aux/IAA* genes. Noteworthy findings include the variability in amino acid length, molecular weight, and isoelectric point, indicative of functional diversity within the gene family. According to the results, three genes, SpoIAA17, SpoIAA18, and SpoIAA19, shared identical sequences from Sp75 genome (Spo08496.1 IDs).

**Table 1:** The list of *SpoIAA* genes along with pertinent details, including Gene Name, Gene ID, Chromosome (Chr), Physical position on the genome, Amino acid length, Molecular weight (Da), Isoelectric Point (PI), and Sp75 ID

Gene Name	Gene ID	Chr	Physical position on the genome	Amino acid length	Molecular weight (Da)	PI	Sp75 ID
SpoIAA1	SOV1g012130.1	1	63,404,212–63,413,398	179	19238.65	9.63	Spo05025.1
SpoIAA2	SOV2g010000.1	2	43,735,470–43,742,680	195	21990.85	9.07	Spo22004.1
SpoIAA3	SOV2g016580.1	2	72,004,330–72,007,547	283	31254.83	5.31	Spo06464.1
SpoIAA4	SOV2g019920.1	2	81,229,675–81,233,361	266	30760.45	5.75	Spo04733.1
SpoIAA5	SOV3g014320.1	3	20,654,170–20,659,912	228	25680.6	6.03	Spo09456.1
SpoIAA6	SOV3g019720.1	3	35,680,617–35,722,170	1099	120574.88	6.27	Spo18975.1
SpoIAA7	SOV3g022300.1	3	44,669,081–44,673,585	237	26560.55	5.79	Spo15863.1
SpoIAA8	SOV3g032050.1	3	89,788,073–89,789,001	160	18222.59	5.17	Spo00063.1
SpoIAA9	SOV4g047550.1	4	169,187,727–169,190,366	117	13482.03	9.62	-
SpoIAA10	SOV4g048620.1	4	171,297,661–171,299,548	205	23517.75	5.75	Spo24603.1
SpoIAA11	SOV4g048640.1	4	171,364,558–171,370,743	186	20893.95	6.34	Spo24602.1
SpoIAA12	SOV4g053610.1	4	180,195,128–180,199,147	339	36677.09	8.78	Spo03500.1
SpoIAA13	SOV4g057440.1	4	185,981,998–185,986,153	306	33386.25	6.83	Spo23123.1
SpoIAA14	SOV4g015600.1	4	36,291,989–36,295,417	288	31004.06	7.56	Spo08497.1
SpoIAA15	SOV4g015640.1	4	36,558,016–36,559,400	183	20758.9	7.57	Spo08494.1
SpoIAA16	SOV4g015670.1	4	36,636,966–36,637,963	81	9198.09	5.3	Spo08499.1
SpoIAA17	SOV4g015680.1	4	36,644,049–36,645,068	167	19234.78	5.82	Spo08496.1
SpoIAA18	SOV4g015720.1	4	36,980,450–36,981,473	167	19202.72	5.82	Spo08496.1
SpoIAA19	SOV4g015730.1	4	36,986,655–36,987,746	238	26898.61	4.39	Spo08496.1
SpoIAA20	SOV5g017190.1	5	26,983,436–26,986,051	199	22076.16	6.43	Spo22710.1
SpoIAA21	SOV5g002600.1	5	3,098,391–3,102,902	261	28367.5	8.65	Spo02286.1
SpoIAA22	SOV5g002610.1	5	3,134,425–3,136,469	196	22013.64	5.8	Spo02371.1
SpoIAA23	SOV6g033740.1	6	128,566,056–128,570,184	318	34411.44	5.95	Spo23507.1
SpoIAA24	SOV6g013400.1	6	54,504,687–54,508,462	226	25163.86	8.72	Spo22710.1

This suggests a possible repetition or redundancy in the annotation or identification of these genes, as they are linked to the same Sp75 ID. While the gene names and physical positions on the genome were distinct for SpoIAA17, SpoIAA18, and SpoIAA19, identical sequences and the shared Sp75 ID suggested a need for further investigation or clarification in the gene annotation or identification process. Chromosomal distribution analysis revealed a dispersed pattern across various chromosomes. Chromosome 1, for example, hosts SpoIAA1, characterized by a relatively moderate amino acid length of 179, a molecular weight of 19238.65 Da, and an PI of 9.63. Meanwhile, Chromosome 6 accommodates SpoIAA23, distinguished by an extended amino acid sequence of 318, a higher molecular weight of 34411.44 Da, and a PI of 5.95. Overall, chromosome 4 and 1 harbored the highest (11 genes) and lowest (1 gene) number of *Aux/IAA* genes, respectively. The variability among the identified *Aux/IAA* genes was further underscored by the unique attributes of individual genes. Notably, SpoIAA6 stands out with an exceptionally long amino acid sequence of 1099 and a correspondingly high molecular weight of 120574.88 Da. In contrast, SpoIAA16 exhibits the smallest amino acid length (81) and the lowest molecular weight (9198.09 Da) among the identified genes. This range in gene length and molecular weight implies functional diversity within the *Aux/IAA* gene family, potentially indicative of specific roles and interactions.

### Gene structure and conserved motif analysis of SpIAA genes

**Fig. 1:**
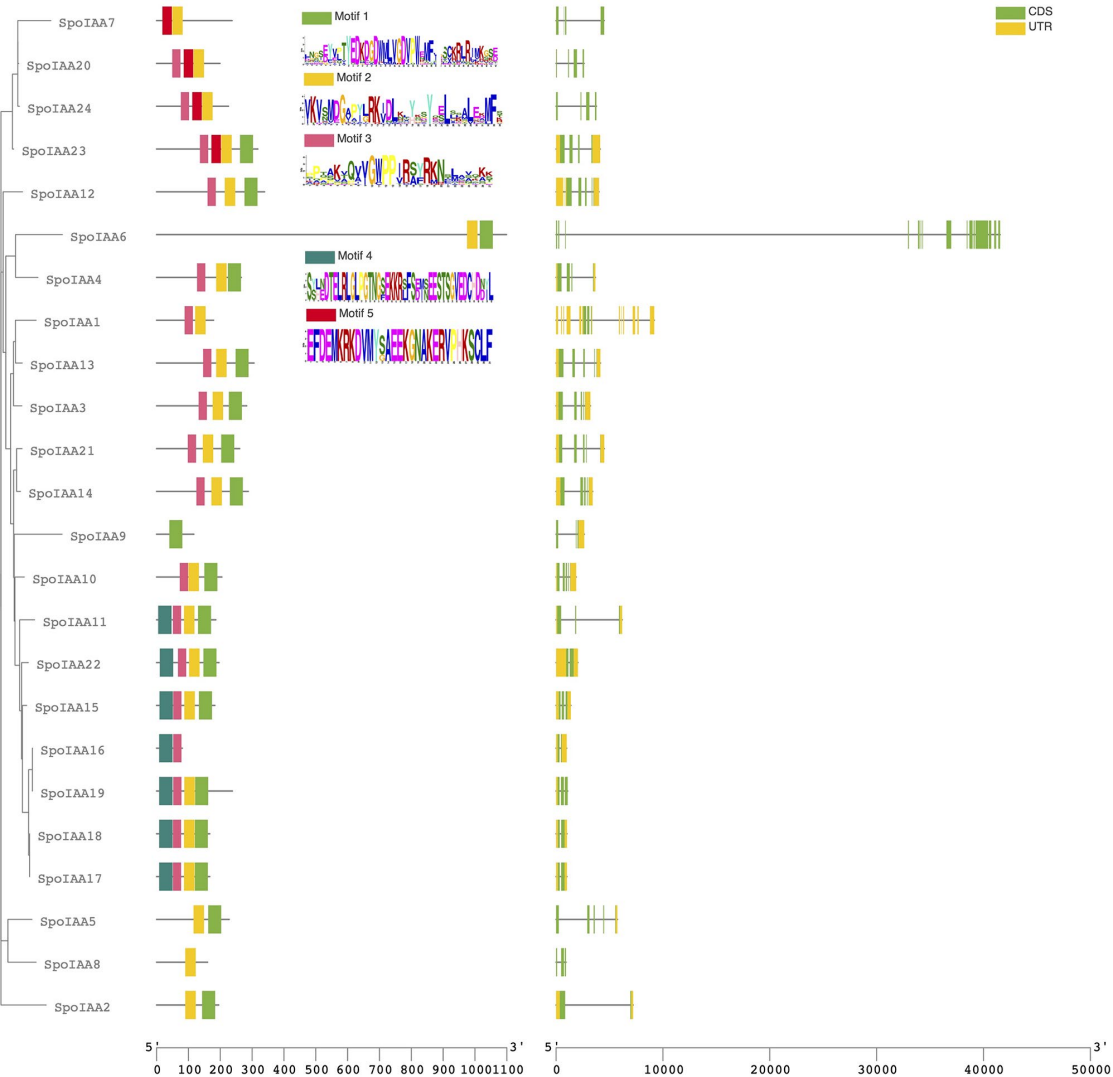
Gene structure and conserved motif analysis of spinach IAAs according to a phylogenetic relationship

The future analysis aimed to assess and compare the characteristics of coding sequences (CDS) and untranslated regions (UTR) among *Aux/IAA* genes in spinach. The investigation revealed notable variability in the number of exons, CDS regions, and UTR regions across the gene set (Fig. 1). According to the results, the number of exons in spinach *Aux/IAA* genes ranged from 3 to 15. Specifically, gene number 6 (SpIAA6) exhibited the highest number of exons (15), whereas the lowest exon count (3) was observed in SpIAA7 and 8. In terms of CDS regions, the most extensive coding was identified in gene number 6, while the least coding regions (2) were present in SpIAA1, 2, 16, 17, 18, and 22. The analysis further unveiled variations in UTR, with the highest number of non-coding regions (11) observed in SpIAA1. In contrast, several SpIAA genes, including numbers 6, 7, 8, 20, and 24, displayed no UTR, indicating a distinctive structural pattern. SpIAA5 exhibited a unique profile with only one non-coding region.

Additionally, we used MEME software to identify five fully conserved domains among Aux/IAA family members in spinach, indicating functional similarities. The analysis revealed variable numbers of motifs across different genes (Fig. 1). Notably, two motifs were identified in SpoIAA1, SpoIAA2, SpoIAA5, SpoIAA6, SpoIAA7, SpoIAA16, while SpoIAA8 and SpoIAA9 had one motif each. SpoIAA10, SpoIAA11, SpoIAA12, SpoIAA13, SpoIAA14, SpoIAA20, SpoIAA21, SpoIAA24 exhibited three motifs, while SpoIAA15, SpoIAA17, SpoIAA18, SpoIAA19, and SpoIAA22 had four motifs. Furthermore, SpoIAA23 displayed the presence of four motifs. The differences in motif numbers among various *Aux/IAA* genes show the variety in motif makeup, which might suggest different functions within the auxin signaling process. The examination of amino acid conservation within these domains unveiled a high frequency of specific residues at consistent positions across sequences, underscoring significant conservation. These results highlighted the presence of conserved DDxxD and KR motifs in Aux/IAA family members. Notably, the KR motif, containing lysine and arginine, was universally shared by all domains, accompanied by a conserved DDxxD motif upstream. Further exploration of specific domains elucidated distinctive amino acid conservation patterns within the KR motifs, emphasizing the importance of these motifs in the structural and functional integrity of Aux/IAA proteins.

### Cis-elements in the promoters of *SpIAA* genes

**Fig. 2:**
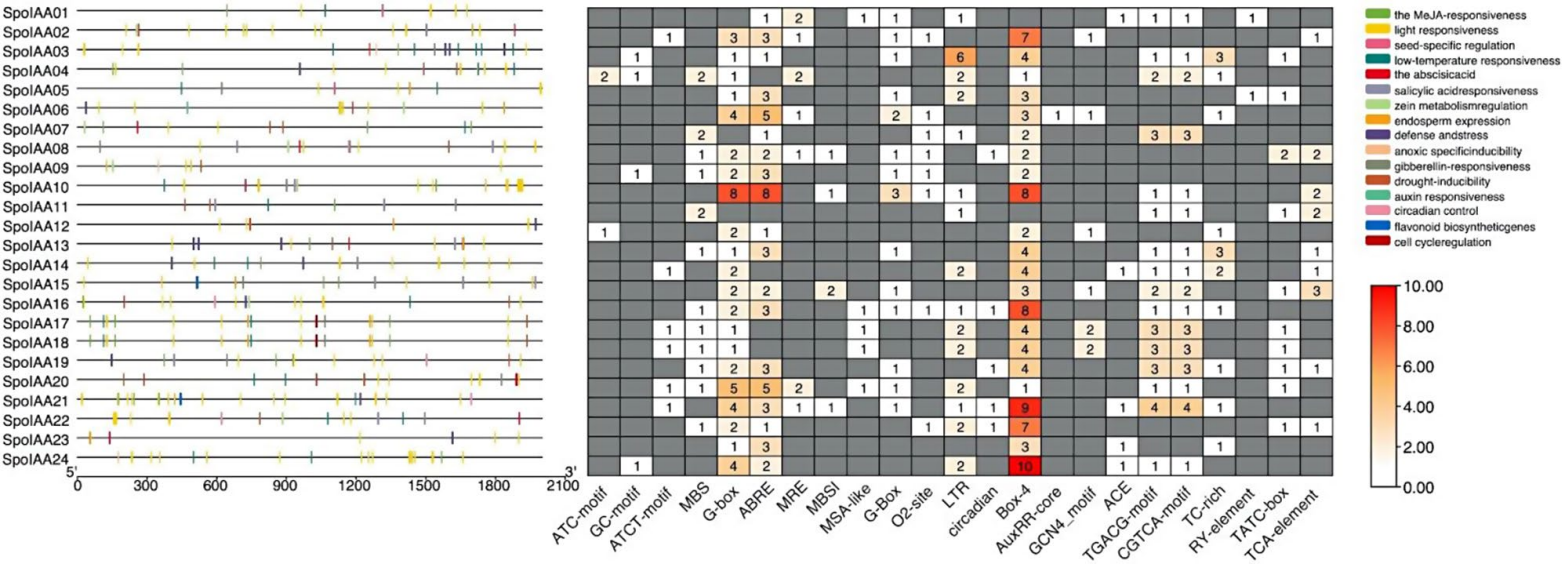
The heatmap illustrates the distribution of cis-acting elements within the 2000 bp promoter region of each *SpoIAA* gene. Different colored boxes on the right side of the heatmap represent various cis-acting elements, each with distinct functional roles

​​The regulatory landscape of *IAA* genes discovered from spinach was investigated by analyzing cis-elements in their respective promoter regions using the PlantCARE Database. An extensive analysis unveiled a wide range of cis-elements linked to various biological processes (Fig. 2). Notably, some elements were associated with hormone responsiveness, such as CGTCA-motif, TGACG-motif, TCA-element, and G-box, which were abundant. These elements were mainly connected to responses to abscisic acid (ABA) and methyl jasmonic acid (MEJA) induced stresses. Furthermore, ABRE elements, recognized for their involvement in ABA responsiveness, were identified, indicating the participation of *IAA* genes in ABA-mediated responses. Additionally, elements related to environmental stresses, including ARE, GC-motif, TC-rich repeats, LTR, MSA-like, and MBS, were found, with ARE and GC-motifs displaying significant abundance and association with anaerobic conditions. Moreover, developmental-related elements like CAT-box, GCN4-motif, and O2-site were detected, suggesting roles in meristem growth, endosperm development, and zein metabolism. The identification of circadian-related elements highlights the potential engagement of *IAA* genes in circadian regulation, suggesting their involvement in the plant’s rhythmic physiological processes. In addition, cis-elements associated with low-temperature responsiveness, such as LTR, were also identified, indicating a potential role for *IAA* genes in cold stress adaptation. Moreover, elements related to light responsiveness, including MRE, Box-4, and ATCT-motif, exhibited high representation, suggesting their involvement in regulating IAA gene expression in response to light cues.

### Phylogenetic analysis of *Aux/IAAs*

**Fig. 3:**
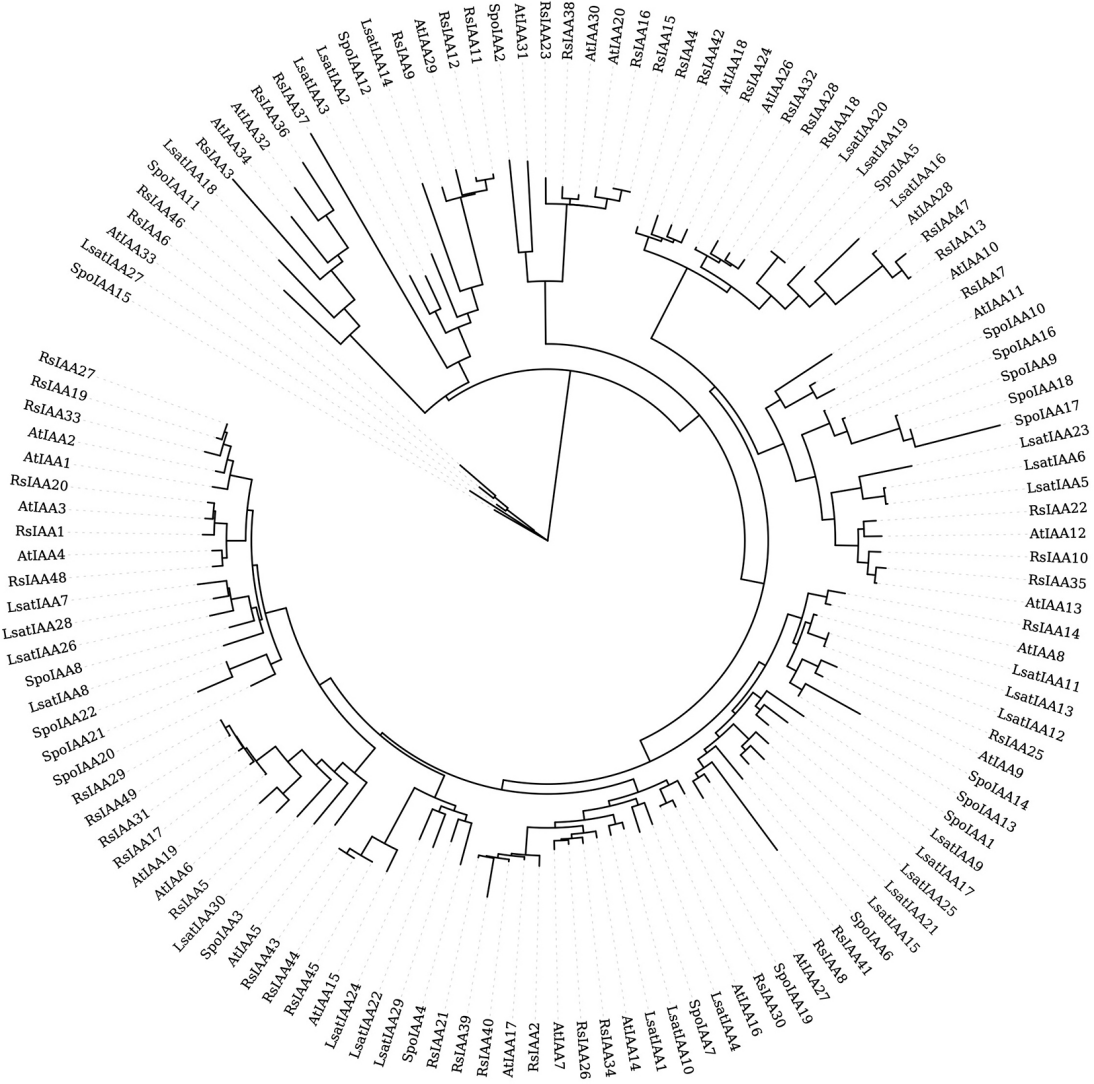
Phylogenetic tree of Aux/IAA proteins from spinach, Arabidopsis, radish and lettuce

The phylogenetic tree, constructed from the amino acid sequences of *Aux/IAA* genes, revealed ten distinct clades (Fig. 3), each representing a unique evolutionary lineage or grouping of *Aux/IAA* genes across radish (Rs), lettuce (Lsat), spinach (Spo), and Arabidopsis (At). Notably, *SpoIAA2* demonstrated close evolution to *AtIAA31*, suggesting a conserved lineage between these genes. Additionally, a subclade comprising radish genes (*RsIAA46* and *RsIAA6*), *LsatIAA27*, along with *AtIAA33*, indicated a potential shared evolutionary history. Similarly, spinach-specific genes, such as *SpoIAA1* and *SpoIAA3*, exhibited distinct placements within the phylogenetic tree, suggesting shared evolutionary origins or potentially specialized roles within the auxin response in spinach. Furthermore, *SpoIAA9*, *SpoIAA17*, and *SpoIAA18*, forming a well-defined clade, suggested either gene duplication events or functional conservation among these spinach-specific genes. Likewise, *SpoIAA13* and *SpoIAA14*, positioned closely within the same clade, displayed a consistent clustering pattern, implying shared genetic ancestry and potential functional similarities. This proximity in the phylogenetic tree indicated a more recent divergence or a common evolutionary trajectory, suggestive of a potential functional synergy in auxin signaling pathways within spinach. Their juxtaposition within the same clade underscored their close genetic relationship, prompting further exploration into their shared or distinct roles in spinach development, growth, and responses to environmental stimuli. Furthermore, the juxtaposition of *SpoIAA20*, *SpoIAA21*, and *SpoIAA22* within the same clade underscored their interconnected evolutionary history, justifying further exploration into their contributions to spinach development, growth, and responses to environmental cues. The unique positioning of *SpoIAA15* within the tree suggested specific evolutionary adaptations or divergences, leading to its distinct role within the auxin response network in spinach. Consistently placing within its designated clade implied a different evolutionary history with specific *Aux/IAA* genes from other plant species, indicating potential distinct functional or regulatory roles in auxin signaling pathways.

### Gene expression

**Fig. 4:**
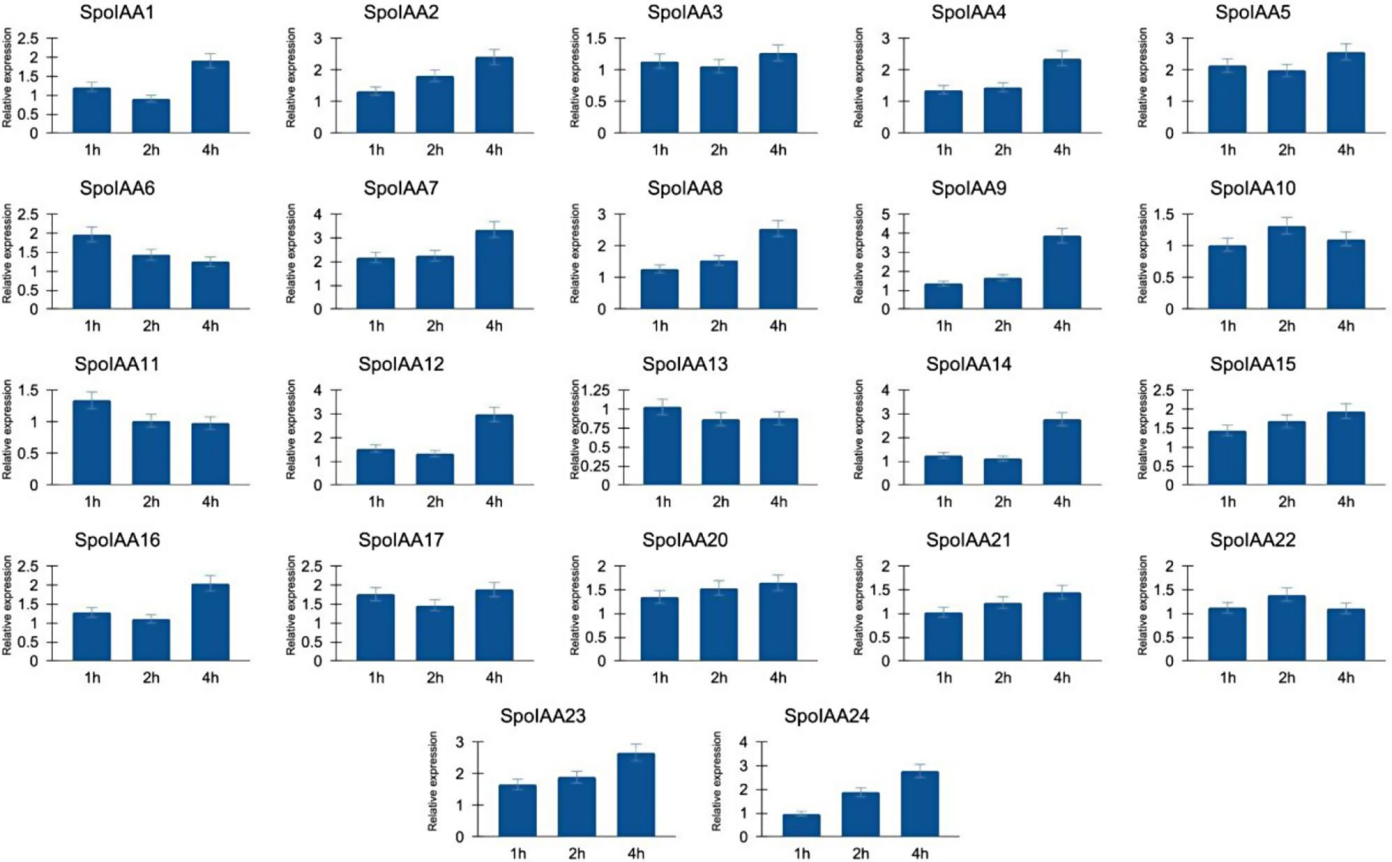
The expression profiles of SpoIAAs in response to NAA treatment were evaluated through qRT-PCR analyses

The expression patterns of 24 *SpoIAA* genes were investigated under the influence of NAA treatment at three distinct time points (1 h, 2 h, and 4 h). The average expression levels for each gene across three replicate samples at each time point are presented in Fig. 4. According to the results, several genes exhibited distinct temporal expression patterns. Notably, *SpoIAA5* displayed a substantial increase in expression at 2 h post-NAA treatment, followed by a moderate decrease at 4 h. In contrast, *SpoIAA7* and *SpoIAA9* demonstrated a continuous rise in expression levels, peaking at the 4-hour time point. These observations suggest that the influence of NAA on gene expression is both gene-specific and time-dependent. Some genes, such as *SpoIAA1*, *SpoIAA3*, and *SpoIAA10*, showed a rapid response to NAA treatment with increased expression at the 1-hour time point, suggesting their involvement in the early stages of auxin signaling. Conversely, genes like *SpoIAA11* and *SpoIAA13* exhibited a transient response, with an initial increase at 1 h followed by a decline at subsequent time points. This transient behavior implied a rapid but temporary modulation of gene expression in these instances. On the other hand, *SpoIAA15* and *SpoIAA17* displayed sustained increases in expression levels, maintaining elevated levels throughout the observation period. These genes may play roles in mediating longer-term responses to auxin. While some genes, such as *SpoIAA2* and *SpoIAA16*, showed consistent increases in expression across all time points, others, like *SpoIAA12* and *SpoIAA21*, exhibited more variable responses, suggesting intricate regulatory mechanisms. Interestingly, *SpoIAA24* exhibited a continuous increase in expression, peaking at 4 h, while *SpoIAA10* displayed a decrease in expression during the same period.

## Discussion

The comprehensive identification and characterization of *Aux/IAA* genes in spinach, as presented in this study, contribute valuable insights into the intricate regulatory network of auxin signaling. Our systematic approach involved utilizing two spinach genomes, Sp75 and Monoe-Viroflay, to ensure a comprehensive survey of the *Aux/IAA* gene family. The employment of HMM and BLAST searches against *A. thaliana* Aux/IAA protein sequences facilitated the precise identification of 24 definitive *Aux/IAA* genes. In comparison, other extensively studied plant species, such as *A. thaliana*, exhibited a rich assortment of gene families. Notably, the Arabidopsis genome encompasses 29 *Aux/IAA* genes [28], *Brassica rapa* boasts 89 *AUX/IAA* genes [29], *Brassica napus* features 119 *AUX/IAA* genes [30], *Hordeum vulgare* contains 36 *AUX/IAA* genes [31], and *Cucumis sativus* harbors 29 *AUX/IAA* genes. Additionally, rice and maize each contain 31 *AUX/IAA* genes [20, 22], Sorghum bicolor comprises 26 members [23], and poplar comprises 35 genes [24], collectively contributing to the intricate regulatory network governing auxin responses. A comparative analysis of *Aux/IAA* gene numbers between spinach and other species reveals both similarities and distinctions. This highlights the evolutionary divergence and specialization inherent within these plant species. The observed differences in the number of *Aux/IAA* genes among plant species can be elucidated through a combination of evolutionary, genomic, and functional factors. Evolutionary processes, including gene duplication, divergence, and loss, contribute to the dynamic nature of gene families across plant lineages. Whole-genome or segmental duplication events play a role in expanding the *Aux/IAA* gene repertoire, allowing for functional diversification within specific species. Functional specialization, driven by the unique developmental processes of each plant, may influence the selection pressure on *Aux/IAA* genes, leading to variations in gene numbers. Furthermore, the adaptation of plants to specific environmental conditions and ecological niches can drive the need for distinct *Aux/IAA* gene sets. Genome assembly and annotation discrepancies, coupled with species-specific requirements, also contribute to reported variations. The observed *Aux/IAA* gene numbers reflect the complex interplay between evolutionary dynamics, selective pressures, and the specific biological needs of individual plant species. This interplay is crucial in modulating auxin signaling for optimal growth and development [2, 32, 33].

The identified *Aux/IAA* genes exhibited considerable diversity in terms of amino acid length, molecular weight, isoelectric point, and motif structure. Such diversity within the gene family, as indicated by the distinctive attributes of individual genes, suggests potential specific roles in mediating auxin responses, such as growth regulation and stress response. Chromosomal organization, synteny, and gene density can influence the distribution and abundance of *Aux/IAA* genes within a genome. Different plant genomes exhibit varying degrees of compactness, repetitive elements, and structural variations, which could account for discrepancies in *Aux/IAA* gene counts. Moreover, recent findings suggest that long terminal repeat retrotransposons (LTR-RTs) contribute to the amplification of *Aux/IAA* genes in grasses like rice and sorghum. Functional redundancy and specialization among *Aux/IAA* gene family members can impact their retention and conservation throughout evolution. While some *Aux/IAA* genes maintain similar functions and thus undergo purifying selection, others accumulate mutations resulting in novel functions or sub-functionalization [32, 34].

In further investigation, we conducted a thorough examination of cis-regulatory elements within the promoters of *SpoIAA* genes. Our analysis revealed associations with various aspects of plant growth, development, and stress responses, including hormonal responses to ABA and MeJA, as well as responses to low temperature and light cues. These findings contribute to our understanding of the molecular mechanisms underlying IAA genes, as well as provide insights into their potential biological roles, building upon previous research into cellular signaling [35–37]. Identifying domains contributes to a deeper understanding of *Aux/IAA* gene function. In our study, we recognized familiar domains such as GWPPV/I, enhancing our comprehension of gene roles. The core sequence of Domain III containing GWPPV/I serves as the target site for ubiquitination degradation of Aux/IAA proteins through interaction with the TIR1 component of the SCFTIR1 complex [31]. In Aux/IAA proteins like *AtIAA20* and *AtIAA30*, the absence of Domain II prolongs protein half-life compared to canonical Aux/IAA proteins, disrupting auxin physiology and resulting in auxin-related aberrant phenotypes [38]. Previous research indicates that non-canonical Aux/IAA proteins lacking Domain II evade degradation by the SCFTIR1 complex and are stabilized through phosphorylation under high auxin levels [39, 40].

In our investigation, we identified *SpoIAA2*, 5, 6, 7, 8, and 9, which lack Domain II, suggesting they may function similarly to previously reported non-canonical IAAs. Additionally, mutations affecting the conserved motif GWPPV/I within Domain II, as seen in *AtIAA31*, *OsIAA4*, *OsIAA10*, and various Arabidopsis mutants (shy2–2, shy2–3, iaa18-1, arx2-1, arx3-1, arx3-3), stabilize the protein and result in typical auxin-related aberrant phenotypes, including dwarfism and altered tiller angles [38, 41, 42]. Domain II plays a pivotal role in facilitating both homo- and hetero-dimerization among Aux/IAA proteins and ARFs, thereby suppressing the transcription of auxin-responsive genes [43, 44]. In our investigation, we observed the absence of Domain II in *SpoIAA6* and 9.

The phylogenetic tree analysis of the *Aux/IAA* gene family in spinach, coupled with expression data at different time points (1 h, 2 h, and 4 h), provided valuable insights into the potential functions of individual members. For example, predicting the function of *SpoIAA8*, 20, 21, and 22 in spinach based on the known function of *Aux/IAA*1, 2, and 3 in Arabidopsis involves considering the conserved nature of *Aux/IAA* genes and their functional domains. *Aux/IAA1* acts as a transcriptional repressor that interacts with auxin response factors (ARFs) to modulate the expression of early auxin response genes. When auxin levels increase, *Aux/IAA1* is rapidly degraded, releasing ARFs to activate or repress downstream target genes involved in various aspects of plant growth and development, such as cell division, elongation, and differentiation. Therefore, *Aux/IAA1* is critical for maintaining proper auxin responses during plant growth and development [45, 46]. *Aux/IAA3* in Arabidopsis is involved in diverse auxin-related processes, such as apical dominance, root elongation, adventitious rooting, and root gravitropism [41, 47]. *Aux/IAA2* plays crucial roles in regulating various developmental processes in plants, including embryo development, lateral root initiation and elongation, hypocotyl growth, tropisms, flower organ development, and other processes. Specifically, when auxin levels change dynamically in space and time, they can trigger gene reprogramming precisely and rapidly, requiring auxin early response genes like *Aux/IAA*. Additionally, *Aux/IAA2* has a close genetic relationship with *SlAux/IAA26* of tomato and *MdAux/IAA26* of apple. When *MdAux/IAA2* was overexpressed in apple flesh callus via *Agrobacterium*-mediated gene transformation, it affected the transcription level of *MdAux/IAA2*, suggesting a role in regulating other abiotic stresses [48]. Given the close phylogenetic relationship between AtIAA1, 2, and 3 and SpoIAA8, 20, 21, and 22, it is plausible to speculate that these genes may also play roles in similar auxin-mediated developmental processes in spinach. The phylogenetic analysis suggests a close evolutionary relationship between *Aux/IAA20* and SpoIAA2, implying that these two genes share a common ancestor and may have conserved functions in auxin signaling. Based on the information provided, *AUX/IAA20* in Arabidopsis is a noncanonical Aux/IAA protein that lacks domain II and is long-lived compared to the canonical IAA17 protein. When overexpressed in Arabidopsis using the 35 S promoter, IAA20 OX shows low root apical meristem (RAM) activity, resulting in reduced meristematic activity in roots. Additionally, IAA20 and IAA30 are early auxin-inducible and expressed in the root apical meristem, suggesting a potential role in maintaining the stem cell niche of the root by regulating the activity of MP/ARF5 and NPH4/ARF7 [49]. Therefore, it is reasonable to hypothesize that SpoIAA14 in spinach may also participate in similar auxin-mediated processes. Given the close phylogenetic relationship between *Aux/IAA5* and 6 from Arabidopsis and SpoIAA3 in spinach, it is reasonable to predict that SpoIAA3 may share functional similarities with *Aux/IAA6* in regulating auxin responses. Therefore, it is plausible that SpoIAA3 in spinach may also contribute to similar processes, potentially influencing root development, tropisms, or other auxin-mediated responses. However, recent characterization of the mutants identified more precise functions in the response to environmental stresses for IAA5, and IAA6 [50–52]. In Arabidopsis, *Aux/IAA12*, also known as SOLITARY ROOT (SLR), plays a key role in regulating various aspects of plant growth and development by modulating auxin signaling. Specifically, *Aux/IAA12* is involved in root development. Mutations in the *Aux/IAA12* gene result in the solitary root phenotype, where the plant forms only a single primary root instead of the normal branched root system. This suggests that *Aux/IAA12* is a negative regulator of lateral root development. The protein is rapidly degraded in response to auxin, allowing ARFs to activate the expression of auxin-responsive genes involved in lateral root initiation [53–55]. *Aux/IAA11* specifically has been shown to be involved in the acclimation to chloroplast-specific stresses and integrates this pathway with auxin signaling [56]. The provided phylogenetic tree indicated that *Aux/IAA10* and 12 from Arabidopsis are close to SpoIAA9, 10, 16, 17, and 18 in spinach. Therefore, a hypothesis can be formulated that these genes in spinach may share some functional characteristics with those in Arabidopsis, possibly participating in root development. Based on the provided information about *AUX/IAA7* in Arabidopsis, which negatively regulates the auxin signaling pathway and exhibits severe growth and development defects when mutated [57], we can predict the potential function of SpoIAA7 and 19 in spinach. Given the close relationship observed in the phylogenetic tree between *Aux/IAA7* from Arabidopsis and SpoIAA7 and 19 in spinach, it is reasonable to speculate that these genes may have a similar role in negatively regulating the auxin signaling pathway in spinach. If these genes function similarly to *Aux/IAA7*, mutations in conserved motifs, particularly in domain II, might result in growth and developmental defects similar to those observed in the *Aux/IAA*7 mutant of Arabidopsis [57]. This could include inhibited stem elongation, leaf curl, slow root growth, fewer lateral roots, and potentially altered gravitropism. The impaired degradation of SpoIAA7 and 19, similar to *Aux/IAA7*, might require higher auxin concentrations to trigger degradation, leading to reduced ARF-mediated gene transcription and, consequently, restricted cell expansion and elongation. Moreover, if SpoIAA7 and 19 are involved in auxin-mediated processes, it may influence cell wall composition, activate cell wall-related gene expression, and contribute to apoplastic acidification, similar to the effects described for auxin in Arabidopsis. These processes collectively contribute to the regulation of plant growth, development, and architecture by controlling cell proliferation, expansion, elongation, and differentiation. Nevertheless, it is important to note that experimental validation is needed to confirm the specific functions of SpoIAA7 and 19 in spinach and to determine whether its role aligns with the predicted functions based on the phylogenetic relationship with *Aux/IAA7* in Arabidopsis.

## Conclusion

In conclusion, this comprehensive investigation systematically identified and characterized the *Aux/IAA* gene family in the spinach genome, particularly in the Sp75 and Monoe-Viroflay genomes. Through a meticulous approach involving bioinformatics tools and databases, a total of 24 definitive *Aux/IAA* genes were identified, each exhibiting diverse characteristics in terms of amino acid length, molecular weight, and isoelectric point. The phylogenetic analysis revealed distinct clades, highlighting evolutionary relationships among *Aux/IAA* genes from spinach, Arabidopsis, radish, and lettuce. The structural analysis, including gene structure and conserved motif analysis, provided insights into the variability in coding sequences and untranslated regions across the *Aux/IAA* gene set. The expression analysis under NAA treatment at different time points revealed gene-specific and time-dependent patterns, shedding light on the dynamic regulation of *Aux/IAA* genes in response to auxin. Additionally, the phylogenetic tree analysis coupled with expression data presented potential functional roles for specific *Aux/IAA* genes in spinach development and auxin-mediated responses. This study contributes valuable insights into the intricate regulatory network of auxin signaling in spinach, providing a theoretical foundation for future investigations into the precise functions and regulatory mechanisms of *Aux/IAA* genes in this important plant species. Indeed, the identification of these genes lays a foundation for comprehending hormone-related molecular mechanisms and offers a valuable genetic resource for molecular breeding, potentially facilitating advancements in agricultural practices and crop improvement strategies.

## Methods

### Identification of *Aux/IAA* gene family in spinach

In this study, we systematically identified the *Aux/IAA* genes in the spinach genome. Initially, all protein sequences of two spinach genomes, including Sp75 [58] and Monoe-Viroflay [59], were retrieved from the relevant genome database, spinachbase.org [60]. Subsequently, a search for the Aux/IAA domain (PF02309) was performed using the Pfam library of Hidden Markov Model (HMM) profiles, employing the hmmer3 software locally (http://hmmer.org/). Genes corresponding to proteins containing the Aux/IAA domain were then extracted from the spinach genome. Following this, a BLASTP search was executed against *A. thaliana* Aux/IAA protein sequences [18, 19], with a stringent E value threshold (1e^− 10^) to minimize false positives. The obtained non-redundant protein sequences were further validated for the presence of the Aux/IAA domain using the Conserved Domain (CD) search service on the NCBI website. In addition, SMART (Simple Model Architecture Research Tool, http://smart.embl-heidelberg.de) [61] database was used to determine whether any candidate Aux/IAA protein sequences were members of the Aux/IAA family.

### Structural analysis of SpIAAs and orthologous identification

Information on identified *Aux/IAA* genes in spinach was retrieved from the spinach genome database, including sequence ID, and chromosomal location. The genomic positions of each identified *Aux/IAA* gene in the spinach chromosomes were determined through BLAST searches against the genomic sequences of each spinach chromosome. Molecular weights (MW) and isoelectric points (PI) were calculated using the Protparam program on the Expasy website (http://au.expasy.org/tools/protparam.html). For the identification of orthologs in radish [62] and lettuce (*Lactuca sativa* cv Salinas V8, https://genomevolution.org/coge/GenomeInfo.pl?gid=28333), the identified *Aux/IAA* genes from spinach were utilized as queries for BLAST searches against the Radish Genome Database and Lettuce Genome Database, respectively. The orthologous relationships were established based on sequence similarity, and unique names were assigned to the identified genes following the nomenclature conventions for each species. The chromosome mapping *of Aux/IAA* genes in radish and lettuce was performed using the respective GFF files. A similar approach to SpIAA was used to determine whether any candidate Aux/IAA protein sequences were members of the Aux/IAA family.

### Conserved motif and promoter cis-elements analysis

To identify conserved motifs within the SpIAA proteins, we utilized the Multiple Expectation Maximization for Motif Elicitation (MEME) online tool (http://meme.nbcr.net/meme4_1/cgi-bin/meme.cgi) [63]. The analysis parameters were configured as follows: allowing zero or one occurrence per sequence for a single motif, setting the optimal motif width between ≥ 6 and ≤ 50, limiting the maximum number of motifs for identification to nine, and maintaining default values for all other parameters. The identified motifs were further annotated using the SMART program (http://smart.embl-heidelberg.de) and Pfam database (http://pfam.xfam.org/) to provide comprehensive functional annotations. In further investigation, the SpIAA gene promoter sequences, which span 2000 base pairs upstream of the translational start site (ATG), were acquired from the spinach genome. The PlantCARE tool (https://bioinformatics.psb.ugent.be/webtools/plantcare/html/) was utilized to analyze cis-regulatory elements present in these promoter regions. Subsequently, TBtools [64] was employed to generate visual representations of these cis-elements.

### Phylogenetic analysis

Protein sequences corresponding to each gene from all *Aux/IAA* (Spinach, Arabidopsis, Radish, and Lettuce) were compiled into a FASTA-formatted file and subjected to multiple sequence alignment using MUSCLE v3.8.31 [65]. The resulting alignments underwent a rigorous filtration process, removing poorly aligned regions through trimAl v1.4 [66] with parameter settings “-gt 0.7 -st 0.01”. Subsequently, maximum likelihood (ML) phylogenetic trees were constructed for alignment sequence using RAxML v8.2.11 [67], employing the PROTGAMMAAUTO model, and support values were evaluated with 100 bootstrap replicates.

### Plant materials, RNA extraction and qRT-PCR

The Virofly spinach cultivar was planted in a controlled greenhouse at Isfahan University of Technology under specific conditions, maintaining a temperature of 16 °C/14°C day/night, a photoperiod of 16 h/8 h light/dark, and 50% relative humidity. To investigate the effects of NAA (1-Naphthaleneacetic acid) treatment, spinach seedlings at the three-week-old stage with four leaves were subjected to a 5 nM NAA spray. Subsequent sampling occurred at 1 h, 2 h, and 4 h post-spraying. Control seedlings were treated with DMSO (dimethyl sulfoxide) as solvant. To minimize expression variability, each sample represented pooled material from a minimum of three plants. Collected samples were rapidly frozen in liquid nitrogen and stored at − 80 °C until RNA extraction. RNA extraction was performed in triplicate using the DENAzist column RNA isolation kit following the manufacturer’s protocol. The RNA concentration and purity were assessed using a NanoDrop Spectrophotometer and agarose gel. Quantification and cDNA library preparation was performed using isolated RNA samples treated with DNase I enzyme to eliminate genomic DNA contamination. The resulting cDNA served as a template for quantitative real-time polymerase chain reaction (qRT-PCR). Primer design for selected genes (Table 2) was conducted using the Primer3 tool, considering parameters such as melting temperature, self-complementarity, hairpin potential, and primer product sizes. The qRT-PCR experiments were performed in triplicate using a StepOne Real-Time PCR system. The reaction mixture included SYBR Green Master Mix, diluted cDNA, and specific primers. The protocol consisted of an initial denaturation step, followed by 40 cycles of amplification, and concluded with a melting curve program. The expression patterns of bolting and flowering-related genes were quantitatively assessed using the 2 ^− ∆∆Ct^ method [68], with GADPH serving as the internal reference gene. This approach allowed for a rigorous evaluation of the targeted gene expressions at various developmental stages in spinach leaf tissues.

**Table 2:** Gene names and primer sequences used for gene expression assays via qRT-PCR

Gene ID	Forward primer (5′ to 3′)	Reverse primer (5′ to 3′)	Product size (bp)
SpoIAA1	GCTTCTAAACCAGGTGTTCAGG	TAGCAGTCGGAGCTCTATTTCC	175
SpoIAA2	CGTTCGATCTGTCACCGTATAA	TGAAGATCACCAGCAAGAAGAA	209
SpoIAA3	TGACAAGAAGAACACCAAAACG	ATCCACCATTTTCAGACAAACC	218
SpoIAA4	AGCTCTTGTTGGTAGCTTGCTC	ATCCTGAGTCGTTGGACAGATT	153
SpoIAA5	TTCACATTCTCTATCGGCCTCT	TCGTGATGATGAGAAGAAATGC	150
SpoIAA6	CCAGTGAGGAAGAAGAGGAGAA	GCCTCCAAAAACTGTGAAAAAC	226
SpoIAA7	ACCGGATGCTTGTTAGAGATGT	AAGTCCTGCTTTCTTGCTTTTG	186
SpoIAA8	GAGTGGCCATACCTCAAAAGTC	CAAGGCTAGGGTATCCAAGATG	175
SpoIAA9	CAGAAGTCTGGTACGCTGTTGA	TGCTTTGCACTTCTCTCTTCTG	176
SpoIAA10	TCGGATACTTTGGATTTGAACC	TCCATCAACTGCAACCTTTACA	207
SpoIAA11	GAACAAGACCTTCATGTGACCA	CATGCATTCCTTTTCACTACCA	182
SpoIAA12	GAAGGAACACAGTCATCAACCA	GCACAGCTGTAAGAGCAGAAGA	218
SpoIAA13	TGCCTCTATGTGAAGGTTAGCA	AGATCTGCATCCTGTTTTCCAT	194
SpoIAA14	CTCTGAGACGGAAGGTGACTCT	TAGGAGCTGCGGTAACTTTCTC	188
SpoIAA15	TGAACAAGAAAGGAGTTGAGCA	CCATCCAATAACTTGTGCCTTT	182
SpoIAA16	TGGGACGGAGTAATGAAAACT	AGGTAGTCGTCATGGCAATCTT	151
SpoIAA17	CGAATTGAGATTAGGGTTACCG	TCTTGTGCGTTCTTCTTGTGAT	211
SpoIAA20	CTCCTACGGCTAGCAATCAAGT	TTAAGATCGACCTTCCTTCCAA	250
SpoIAA21	TCATGAATGAGAGCAAGCTGAT	ATAGCTCTTGGAGCAAGTCCAG	181
SpoIAA22	ACCTGAGGAAGATTGACCTGAA	ACATCTCCCATGGTACATCTCC	189
SpoIAA23	ACTCGTTAGGGCTAATGTTGGA	AAGGAACATCTCCAACAAGCAT	183
SpoIAA24	AAGGGTAGAAGCAGCAGCTATG	ACGCGCTTAGTACCAGAAGAAG	161

## Data Availability

The datasets (Sp75 and Viroflay genome data) used during the current study are available in Spinachbase.org, and gene expression data available from the corresponding author on reasonable request.
